# Clinical trials in otology and neurotology: state of the science

**DOI:** 10.3389/fneur.2025.1598789

**Published:** 2025-07-24

**Authors:** Lindsay S. Moore, Varun Sagi, Konstantina M. Stankovic

**Affiliations:** ^1^Department of Otolaryngology-Head and Neck Surgery, Stanford University School of Medicine, Stanford, CA, United States; ^2^Department of Neurosurgery, Stanford University School of Medicine, Stanford, CA, United States; ^3^Wu Tsai Neurosciences Institute, Stanford University, Stanford, CA, United States

**Keywords:** otology, neurotology, clinical trials, hearing loss, translational therapies

## Abstract

**Objective:**

To evaluate the current state of interventional clinical trials in otology and neurotology.

**Study design/methods:**

Review of registered clinical trials on ClinicalTrials.gov from January 1st 2019 through May 31st 2025. Interventional trials and those that met keyword criteria for otologic/neurotologic disorders were included. For each study, key characteristics including trial status, trial phase, study design, participants, intervention type, funding source, and results status were collected.

**Setting:**

National database.

**Results:**

Though the number of interventional otologic and neurotologic clinical trials has grown over the past 15 years, in the past 5 years, there has been a stagnation of the steady growth seen in the preceding ten. The greatest proportion of trials were focused on hearing loss, utilized devices, were randomized, and were funded by sources other than industry or the government. Compared to 2008–2018, trends included a shift towards device and procedural interventions for vestibular disorders and a decrease in device trials and increase in behavioral interventions for tinnitus. Emerging areas include novel pharmacological and gene therapies for hearing loss and vestibular schwannoma, but these areas remain gaps and are promising therapeutic avenues that merit further exploration.

**Conclusion:**

Future interval assessments exploring the trends in otologic and neurotologic clinical trials should be performed to identify gaps that offer opportunities for innovation of novel therapies and to monitor the health of the clinical trial environment.

## Introduction

Clinical trials represent the critical step in the translation of innovations forged at the bench to novel therapies utilized at the bedside. Panelists of the American Academy of Otolaryngology—Head and Neck Surgery Foundation (AAO-HNSF) tasked with producing clinical practice guidelines consider randomized controlled trials (RCTs) among the highest level of evidence, and therefore irrefutably impactful in shaping the standards of care for Otolaryngology—Head and Neck Surgery (OHNS) (1).

However, OHNS trials remain underrepresented in OHNS literature (2–4) and constitute a smaller portion of overall clinical trials relative to the burden of OHNS diseases, and this disparity is perhaps widest in the field of otology and neurotology (5). One study of all registered interventional trials from 2007 to 2010 found only 2.7% were OHNS trials, and of those, only 11.1% addressed otologic conditions, yet otologic disorders were the most common outpatient diagnoses encountered by OHNS providers (35.9% of visits) (5).

There exists a paucity of literature specifically exploring clinical trials in otology and neurotology. The only known study to do so characterizes registered otologic clinical trials from 2008 to 2018 and found a steady increase in the total number of trials, driven by a disproportionately large increase in hearing loss trials starting in 2012 (6). Nevertheless, treatment of hearing loss remains largely unchanged and arguably lacking over a decade later. Meanwhile, scientific advances have reshaped the clinical and research landscape of the field, with integration of artificial intelligence into vestibular rehabilitation plans, shifts toward behavioral therapies for tinnitus, and the emergence of genetic therapy trials for hearing loss.

In light of the evolving atmosphere of clinical research, the impact of clinical trials on developing evidence-based practice guidelines, and the lack of otologic clinical trials and literature examining them, we endeavored to provide an update into the state of otologic and neurotologic clinical trials from 2019 to 2025.

## Methods

Data was collected from ClinicalTrials.gov, which is a United States (US) based website and online database of clinical research studies, which also includes studies conducted internationally. All studies registered in the database which met our inclusion criteria were included. The database was queried to include all neurotologic clinical trials from the time period January 1st, 2019 through May 31st, 2025 using relevant search terms (Supplementary methods) within the “condition/disease” portion of the search criteria and filtered studies to include only interventional studies. Two researchers (LSM and VS) reviewed each study description and removed any studies that met keyword criteria but did not have a primary focus on an otologic or neurotologic condition. Each study was sorted into one of six broad otologic condition categories (hearing loss, vestibular disorders, tinnitus, otologic infections, vestibular schwannoma, and other) based on the study description. The “other” category included any studies that did not fall into the aforementioned conditions and primarily involved those that studied middle ear disorders. For intervention type, the ClinicalTrials.gov data sorting algorithm automatically sorted a significant portion of studies into the category of “Other.” To make more meaningful data interpretation, the text component describing the intervention for each “Other” study was independently reviewed (LSM and VS) and sorted into the appropriate category (device, drug, behavioral, procedure, diagnostic test, or genetic).

For each study, we recorded trial status, results status, participants, intervention type, funding source, study design, and, if applicable, inclusion in a food and drug administration (FDA) clinical trial phase (7). When graphically presenting the number of otologic trials each year, only data for 2019–2024 were depicted, given that the 2025 data was not for the complete year. Descriptive statistics were used to analyze categorical variables. Simple linear regression analyses were conducted to evaluate trends. Grubb’s statistical test was used to determine if there were any outliers for the number of trials per year between 2019 and 2024. *p* values <0.05 were deemed significant. Study exempt from IRB approval as no human participant data presented.

## Results

A total of 915 neurotology clinical trials registered on ClinicalTrials.gov during the time period from January 1st 2019 through May 31st 2025, met criteria for inclusion. The general characteristics of these trials are outlined in Supplementary Table 1.

**Figure 1:**
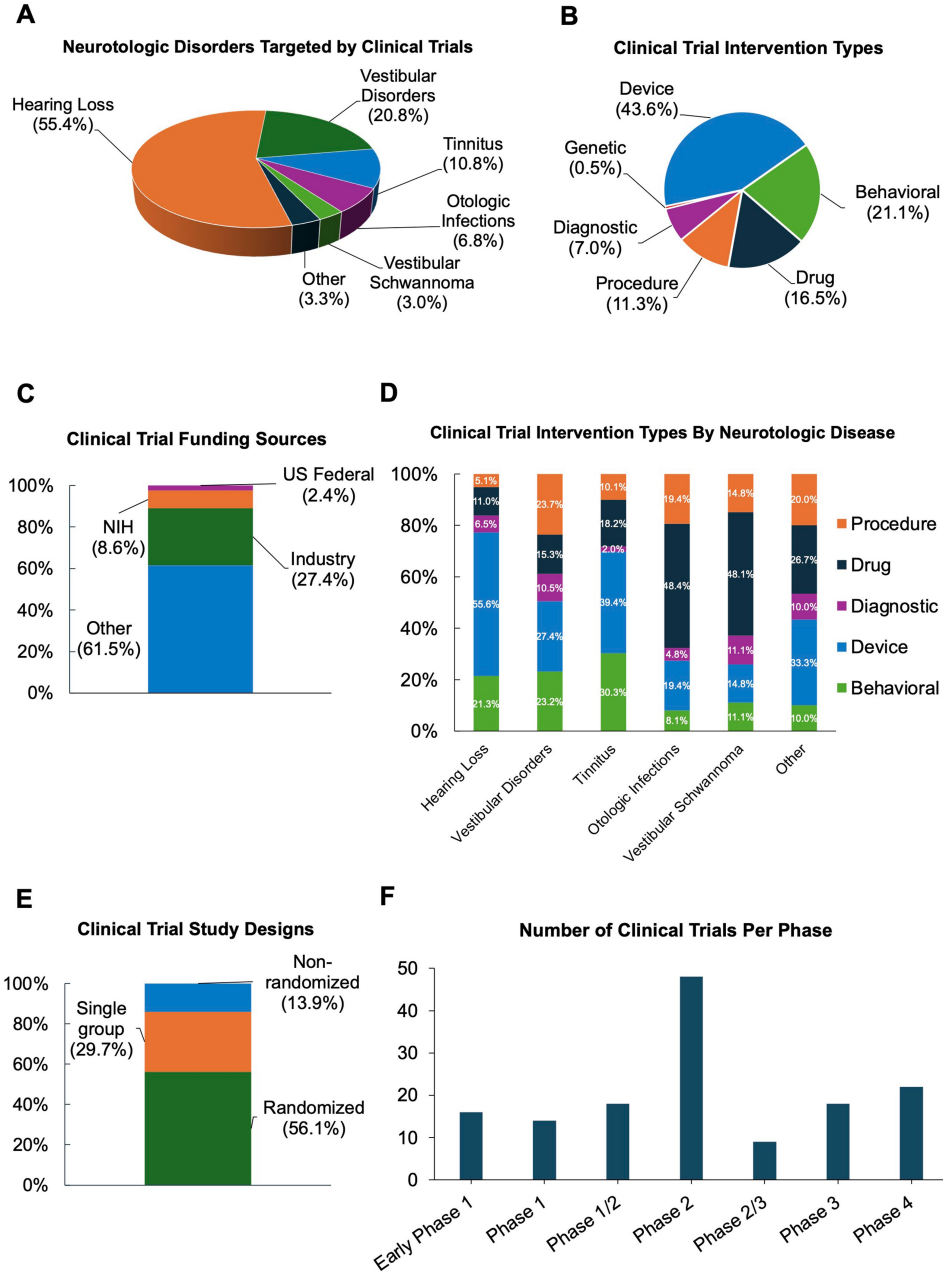
Characteristics of interventional otologic and neurotologic clinical trials from 2019 to 2025. **(A)** Disorders targeted. **(B)** Intervention type. **(C)** Funding sources. **(D)** Intervention type by disorder. **(E)** Study designs. **(F)** Trials by food and drug administration (FDA) clinical trial phase.

Hearing loss (55.4%) was the most common neurotologic condition studied followed by vestibular disorders (20.8%), tinnitus (10.8%), otologic infections (6.8%), and vestibular schwannoma (3.0%) (Figure 1A). For study intervention, device trials were most common (43.6%) followed by behavioral (21.1%), drug (16.5%), procedural (11.3%), diagnostic (7.0%), and genetic (0.5%) interventions (Figure 1B).

Given the inclusion of several international clinical trials, reported funding sources (Figure 1C) varied widely, with the majority (61.5%) of trials listing funding sources as “Other,” which, per ClinicalTrials.gov, includes individuals, universities, and community-based organizations. Universities and hospitals sponsored nearly all trials in our study with “Other” listed as the funding source. Industry (27.4%) was the second most common funding source. Medical device companies were the most frequent industry funders (Supplementary Figure 1). The remainder of studies were funded by the National Institutes of Health (NIH) funding (8.6%) or other United States federal funding (2.4%), which was the US Department of Veteran’s Affairs for all trials in this category within our study (Figure 1C).

Device interventions were most commonly investigated in hearing loss (55.6%) and tinnitus (39.4%) trials (Figure 1D) whereas drug interventions were most frequently tested in otologic infections (48.4%) and vestibular schwannoma (48.1%). The intervention type for trials targeting vestibular disorders and other otologic disorders were more evenly distributed (Figure 1D). Diagnostic tests were the minority of trials for all otologic disorders.

The majority of studies were randomized trials (56.1%); 29.7% of trials had a single experimental arm, and 13.9% were non-randomized (Figure 1E). Only 15% of the trials were listed as being in one of the FDA defined trial phases (7), of these, the majority were in Phase 2 (33.6%), followed by Phase 4 and Phase 3 (14.3 and 12.1%, respectively), and only 10.0% of studies were in Phase 1 (Figure 1F).

**Figure 2:**
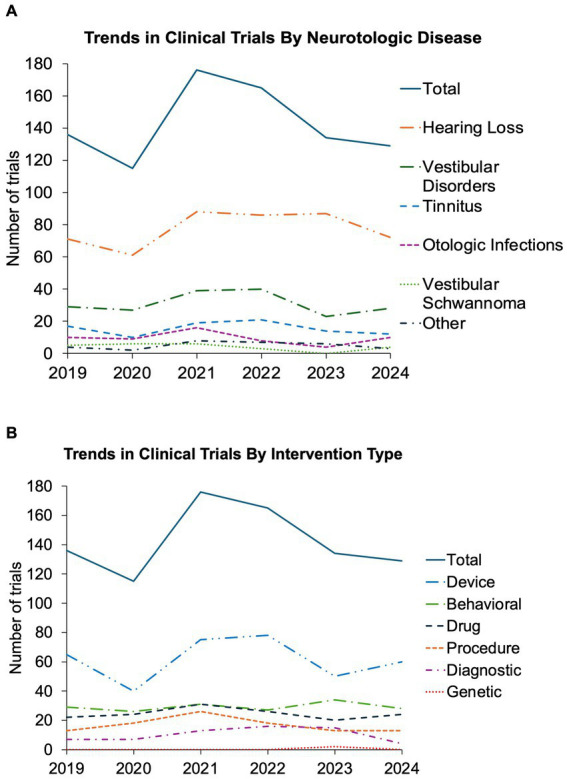
Trends in interventional otologic and neurotologic clinical trials from 2019 to 2024. Trends shown by year from 2019 to 2024 for all otologic and neurotologic clinical trials **(A)** targeting each neurotologic disorder and **(B)** utilizing each intervention type. Solid line represents total number of captured clinical trials for each year.

Overall, our data show strong clinical trial activity within the field of neurotology with more than new 100 trials registered each full year since 2019 (Figures 2A,B). The year with the fewest trials was 2020, followed by the year with the most trials in 2021. Grubb’s test did not find either of these to be statistically significant outliers. The trends in total trials, disease focus, and intervention type have been relatively stable without statistically significant change in the distribution over time (Figures 2A,B). Though not significant, there was a downward trend in the total number of trials, as well as for most interventions and otologic disorders studied after 2021, most notably in 2023. Additional figures outlining funding source, trial design, and inclusion in an FDA trial phase are available in Supplementary Figures 2–7.

## Discussion

### Overall trends in otologic and neurotologic clinical trials

A total of 915 interventional otologic and neurotologic clinical trials met inclusion criteria, comparable to the reported ~774 interventional trials in the preceding 10 years (2008 to 2018) (6), indicating an increase in total clinical trials in the past 5 years compared to the previous decade.

The lowest number of trials was in 2020, likely due to the COVID-19 pandemic (8). There was a strong bounce back in the year 2021 which may reflect easing of pandemic restrictions. There has been a downward trend in the years following, with 2023 reaching near pandemic levels.

### Trends in otologic diagnoses targeted in clinical trials

Similar to the study by Altshuler et al. (6) we also found hearing loss to be most targeted by neurotologic clinical trials, comprising over half (55.4%) of trials. We did not observe the trend of increased hearing loss trials per year seen in the earlier study (6), but we did similarly see the trends in hearing loss trials per year closely mimic those of the overall trial total, consistent with the former taking the majority share of trials. Given the significant prevalence and burden of hearing loss globally (9) and the lack of FDA-approved systemic therapies (other than sodium thiosulfate to prevent cisplatin-induced hearing loss in children) (10), this focus appears merited, though should not come at the cost of clinical research focused on therapies for other neurotologic disorders.

### Trends in clinical trial intervention types

The percentage of studies exploring investigational devices and procedures in 2019–2025 remained roughly the same as during the 2008–2018 interval (6), with device trials maintaining dominance amongst intervention types. Interestingly, this consistent trend of device trials as the lead intervention type for otologic clinical trials is in contrast to trends for clinical trials in the overall field of OHNS, for which multiple sources consistently identified drug trials as the most prevalent (4, 5, 11, 12). However, our findings are in line with previous studies which have found devices to be the most common intervention type in otologic clinical trials (5, 6). Drug trials in OHNS were predominated by head and neck oncologic treatments and medical therapies for allergic and rhinologic disease (4, 5). While medical therapies are a major component of routine management for both fields, otology’s most common disorder, hearing loss, relies heavily on devices (hearing aids and cochlear implants).

The composition of the types of study interventions for clinical trials targeting hearing loss identified in this study closely resembles the findings of the earlier study by Altshuler et al. (6), with devices accounting for over half (55.2%) of trials, most relating to advances in cochlear implants and traditional hearing aids. The percentage of drug trials for hearing loss from 2019 to 2025 was similar to that of 2008–2018. Currently emerging therapies entering trials in increasing numbers include agents proposed to mitigate hair cell oxidative stress, like glutathione peroxidase; manipulate the cell death cascade, like calcineurin antagonists; induce hair cell and/or synapse regeneration, like the *γ*-secretase and glycogen synthase kinase 3 (GSK3)/histone deacetylase (HDAC) inhibitors, or a combination of these mechanisms (13–19). While many of these agents are in ongoing clinical trials, some completed trials have failed to demonstrate efficacy. Nevertheless, the underlying mechanisms of action remain promising, likely prompting the development of future novel compounds designed to work in similar ways (13–19).

A feature not seen in 2008–2018 is the emergence of genetic therapy trials for hearing loss (14–18). Though accounting for only 0.5% of clinical trials, years of development and encouraging preclinical data support the long-awaited arrival of genetic therapies for otologic and neurotologic diseases (14–18). We predict the number of trials in this area will continue to increase and expand beyond congenital hearing loss.

Another interesting trend in trial intervention types between 2008–2018 and 2019–2025 was the shift towards device (from 15.5 to 27.4%) and procedural (from 17.2 to 23.7%) trials in vestibular disorders (6). New devices are implementing augmented reality and machine learning. Notable investigational devices for vestibular disorders seen in recent trials included augmented reality, app-based management aids, and vestibular implants. Several rehabilitation exercises (classified as procedures), as well as cervical manipulation and acupuncture were seen in review of procedural trials for vestibular disorders. Incorporation of artificial intelligence into rehabilitative treatment plans carries exciting potential to provide tailored care targeted to unique patient needs (20–24).

Finally, for exploratory treatments for tinnitus between 2008–2018 and 2019–2025, there was a decrease in device trials (from 50.7 to 39.4%) and an increase in behavioral therapy trials (from 17.7 to 30.3%) (6). Upon review of specific therapies listed for behavioral trials for tinnitus, strategies involving forms of cognitive behavioral therapy, which has previously demonstrated success in improving tolerance and quality of life (25–30), were abundant, as well as mindfulness and wellness counseling, music/sound therapy, and various combinations of these. Many of these therapies leverage technology to facilitate use and compliance, and this trend is likely to continue in the coming years.

### Randomization in otologic and neurotologic clinical trials

Multi-arm, randomized controlled trials offer some of the highest level of evidence used by the AAO-HNSF to develop clinical practice guidelines (1), and randomization is a critical component of rigorous trial design to avoid bias (4, 7). Of otologic and neurotologic trials between 2019 and 2025, 56.1% were randomized, an increase compared to 50.1% for the 2008–2018 interval (6). In several studies exploring clinical trials in OHNS between 2007 and 2018, randomization rates ranged from 54 and 65% (4–6). Overall, this is a commendable improvement for the field. One study published in 1997 searched all articles published in the 10 otolaryngology journals with the highest citation indexes and circulation numbers and found 35 or fewer total RCTs per year between 1989 and 1995, and less then 15 per year between 1961 and 1989 (31). From 1990 and 2003, RCTs comprised only 4–16% of OHNS literature (32, 33), and from 2000 to 2005, only 22% of OHNS RCTs reported valid randomization methods (3).

### Funding sources for otologic and neurotologic clinical trials

Globally, the majority (61.5%) of otologic trials in our study were funded by an unlisted “other” source. While universities and hospitals sponsored nearly all of these trials, it is not possible to discern whether they provided the primary source of funding. Industry funded nearly a third of trials (27.4%), with medical device companies being the most represented. Interestingly, our results showed fewer industry-sponsored trials than was seen in otologic clinical trials from 2008 to 2018 (76.5%) (6), but this may reflect a difference in reporting as no trials are listed as having “other” funding sources despite similar studies within this time frame reporting trials with “other” funding sources (5). Multiple studies have shown that industry-sponsored trials are more likely than non-industry sponsored ones to recommend the interventional agent over placebo or alternative (34–37), and one was unable to explain the greater favorable outcomes for the experimental group by treatment effect or adverse events (34). However, two studies looking specifically at OHNS RCTs found that, in contrast to such findings in other fields of medicine, industry-funded trials were not associated with increased positive findings for experimental agents in our field (3, 4).

As biases related to industry sponsorship are possible, and given the gravity of biased outcomes in RCTs that may guide practice guidelines, physicians must be capable of critically assessing clinical trials, their statistical analyses and outcomes (3, 34–38). Nevertheless, industries remain a key source of critical funding for clinical trials in our field and have enabled the translation of thousands of life-saving interventions (3, 5, 6, 37, 38). The onus is upon surgeons and scientists to practice diligent and transparent reporting, honest disclosures, and prioritize quality trial designs, like appropriate limitation of sponsor support and the use of intention-to-treat analyses (3, 39, 40).

### Clinical trial phases for otologic and neurotologic drug trials and strategies for facilitating efficient translation

Of the 15% of total otologic trials listed in an FDA trial phase, the majority (33.6%) were in Phase 2. Our results coincide roughly with data from OHNS trials between 2007 and 2010 (Phase 2: 24.6%, Phase 3:19.5%, Phase 4:13%) and non-OHNS trials from the same time (Phase 2: 20.6%, Phase 3:15%, Phase 4:13.6%) (5), though both had fewer trials in Phase 2 than otologic trials did in our assessment. It is well-documented that a large percentage of novel drugs showing pre-clinical promise and adequate safety advance to Phase 2 but fail to progress to Phase 3, either due to failure to demonstrate positive results, or, if efficacy is shown, due to the large jump in cost, resources, and time required to conduct a Phase 3 trial (41–45).

The process of developing novel drugs has become increasingly time and cost intensive, hindering the translation of therapies from the bench to the bedside. Strategies must be employed in our field to facilitate success and make this process more expeditious, efficient, and effective (41–45). Phase1/2 trial designs, which can provide safety, efficacy, and dose optimization data, without the added time and resources of two separate trials (46), may be an underutilized strategy in otologic clinical trials (11.5%). Similarly, Phase 2/3 trial designs may increase trial efficiency by allowing for shorter trial durations with smaller sample size (41, 44, 45); however, this approach was even less utilized in otologic clinical trials (6.1%) and may facilitate progression past the Phase 2 “hump” captured in our study. Finally, though the importance of traditional RCTs has already been stated, a discussion of strategies to facilitate the initiation, efficient conduction, and success of clinical trials should include the power of adaptive trial designs (such as platform, basket, and umbrella trials), which are designed to be flexible and responsive to data as they emerge during the trial process (47–50). Similarly, real-world evidence trials collect and analyze data from real world settings, as opposed to traditional randomized controlled trials conducted under controlled conditions (51, 52).

### Study limitations

As with all databases, ClinicalTrials.gov is inherently subject to limitations in design. Free-text entry and open formatting are included in the trial registration process, and data are entered manually by the sponsor or registrant. As such, inaccurate or variability in reporting, capturing, and sorting of data are possible. However, ClinicalTrials.gov remains the most comprehensive and widely used database for clinical trials. Additionally, the National Library of Medicine does a limited review of the study information submitted for inclusion in the study record. Moreover, we performed manual review of the data, cross-confirmed the entries, and re-categorize some data when appropriate, to ensure accuracy.

## Conclusion

Clinical trials are an integral steppingstone between the development of novel therapies and translation to clinical practice. The number of otologic and neurotologic clinical trials have increased overall in the past 15 years, but the number of new interventional trials each year has stagnated in the past 5 years. Emerging areas include gene therapy and drugs with novel mechanisms of action for hearing loss, as well as the integration of artificial intelligence and augmented reality into behavioral therapies for tinnitus and rehabilitation programs for vestibular disorders. Researchers and physicians should strive to continue innovation and advance to testing in clinical trials. Interval assessments exploring the trends specifically in otologic trials should be performed to illuminate promising opportunities, highlight gaps where more studies are needed, and assess the health of the otologic clinical trial environment.
