# Supplementary material for: Clinical trials in otology and neurotology: state of the science
Source: Front Neurol. 2025 Jul 24;16:1598789. doi: 10.3389/fneur.2025.1598789 (PMC12328378; doi:10.3389/fneur.2025.1598789)
Supplement: Supplementary file 1 [file Data_Sheet_1.docx]

**Clinical Trials in Otology and Neurotology: State of the Science**

**Authors:**

Lindsay S. Moore, Varun Sagi, Konstantina M. Stankovic

**Supplemental Methods**

Search terms:

Otology, neurotology, hearing, hearing loss, tinnitus, dizziness, vestibular, vertigo, vestibular schwannoma, neurofibromatosis type 2, otitis, tympanic membrane, ear, vestibulopathy, cochlear, middle ear, inner ear, semicircular canal, ear canal, ear infection, temporal bone, Meniere’s, ototoxic.

**Supplemental Figure Legends**

**Supplemental Figure 1. Sources of industry funding for otologic and neurotologic trials 2019-2025.**

**Supplemental Figure 2. Characteristics of hearing loss trials from 2019-2025. (A)** Funding sources. **(B)** Intervention type by disorder. **(C)** Trials by food and drug administration (FDA) clinical trial phase.

**Supplemental Figure 3. Characteristics of vestibular disorder trials from 2019-2025. (A)** Funding sources. **(B)** Intervention type by disorder. **(C)** Trials by food and drug administration (FDA) clinical trial phase.

**Supplemental Figure 4. Characteristics of tinnitus trials from 2019-2025. (A)** Funding sources. **(B)** Intervention type by disorder. **(C)** Trials by food and drug administration (FDA) clinical trial phase.

**Supplemental Figure 5. Characteristics of otologic infection trials from 2019-2025. (A)** Funding sources. **(B)** Intervention type by disorder. **(C)** Trials by food and drug administration (FDA) clinical trial phase.

**Supplemental Figure 6. Characteristics of vestibular schwannoma trials from 2019-2025. (A)** Funding sources. **(B)** Intervention type by disorder. **(C)** Trials by food and drug administration (FDA) clinical trial phase.

**Supplemental Figure 7. Characteristics of other otologic and neurotologic trials from 2019-2025. (A)** Funding sources. **(B)** Intervention type by disorder. **(C)** Trials by food and drug administration (FDA) clinical trial phase.

**Supplemental Tables**

| **Supplementary Table 1**. Characteristics of Interventional Otologic and Neurotologic Clinical Trials from January 2019 through May 31^st^ 2025. | | | |
| --- | --- | --- | --- |
|  |  | Number (n) | Percentage |
| Trial Status |  |  |  |
|  | Active-Not Recruting | 43 | 4.7% |
|  | Completed | 303 | 33.1% |
|  | Enrolling By Invitation | 31 | 3.4% |
|  | Not Yet Recruiting | 95 | 10.4% |
|  | Recruiting | 291 | 31.8% |
|  | Suspended | 4 | 0.4% |
|  | Terminated | 23 | 2.5% |
|  | Unknown | 90 | 9.8% |
|  | Withdrawn | 35 | 3.8% |
|  | **Total:** | **915** | 100.0% |
| Results Posted | |  |  |
|  | No | 830 | 90.7% |
|  | Yes | 85 | 9.3% |
| Number of Participants | |  |  |
|  | <50 | 478 | 52.2% |
|  | 50-100 | 215 | 23.5% |
|  | 101-150 | 79 | 8.6% |
|  | 151-200 | 31 | 3.4% |
|  | >200 | 109 | 11.9% |
|  | Unknown | 3 | 0.3% |
| Funding Source | |  |  |
|  | Industry | 251 | 27.4% |
|  | NIH | 79 | 8.6% |
|  | Federal (US) | 22 | 2.4% |
|  | Other | 563 | 61.5% |
| Trial Phase |  |  |  |
|  | Early Phase 1 | 16 | 1.7% |
|  | Phase 1 | 14 | 1.5% |
|  | Phase 1/2 | 17 | 1.9% |
|  | Phase 2 | 47 | 5.1% |
|  | Phase 2/3 | 9 | 1.0% |
|  | Phase 3 | 17 | 1.9% |
|  | Phase 4 | 20 | 2.2% |
|  | NA/Not Listed | 775 | 84.7% |
| Otologic Category | |  |  |
| 1 | Hearing Loss | 507 | 55.4% |
| 2 | Vestibular Disorders | 190 | 20.8% |
| 3 | Tinnitus | 99 | 10.8% |
| 4 | Otitis Media/Infection | 62 | 6.8% |
| 5 | Vestibular Schwannoma | 27 | 3.0% |
| 6 | Other | 30 | 3.3% |
| Intervention Type | |  |  |
|  | Behavioral | 124 | 13.6% |
|  | Device | 354 | 38.7% |
|  | Diagnostic Test | 47 | 5.1% |
|  | Drug | 151 | 16.5% |
|  | Genetic | 5 | 0.5% |
|  | Procedure | 61 | 6.7% |
|  | Other/Not Listed | 173 | 18.9% |
| Patient Age |  |  |  |
|  | Child | 92 | 10.1% |
|  | Adult | 58 | 6.3% |
|  | Older Adult | 24 | 2.6% |
|  | Child, Adult | 17 | 1.9% |
|  | Adult, Older Adult | 634 | 69.3% |
|  | Child, Adult, Older Adult | 87 | 9.5% |
|  | Not Listed | 3 | 0.3% |
| Study Design | |  |  |
|  | Single Group | 272 | 29.7% |
|  | Non-Randomized | 127 | 13.9% |
|  | Randomized | 513 | 56.1% |
|  | Other/Not Listed | 3 | 0.3% |
| Year (Start Date) | |  |  |
|  | 2019 | 136 | 14.9% |
|  | 2020 | 115 | 12.6% |
|  | 2021 | 176 | 19.2% |
|  | 2022 | 165 | 18.0% |
|  | 2023 | 134 | 14.6% |
|  | 2024 | 130 | 14.2% |
|  | 2025 | 56 | 6.1% |
|  | Not listed | 3 | 0.3% |
|  |  |  |  |
| Location |  |  |  |
|  | United States | 369 | 40.3% |
|  | Non-United States | 546 | 59.7% |
