# Supplementary figures and images for: Clinical trials in otology and neurotology: state of the science
Source: Front Neurol. 2025 Jul 24;16:1598789. doi: 10.3389/fneur.2025.1598789 (PMC12328378; doi:10.3389/fneur.2025.1598789)

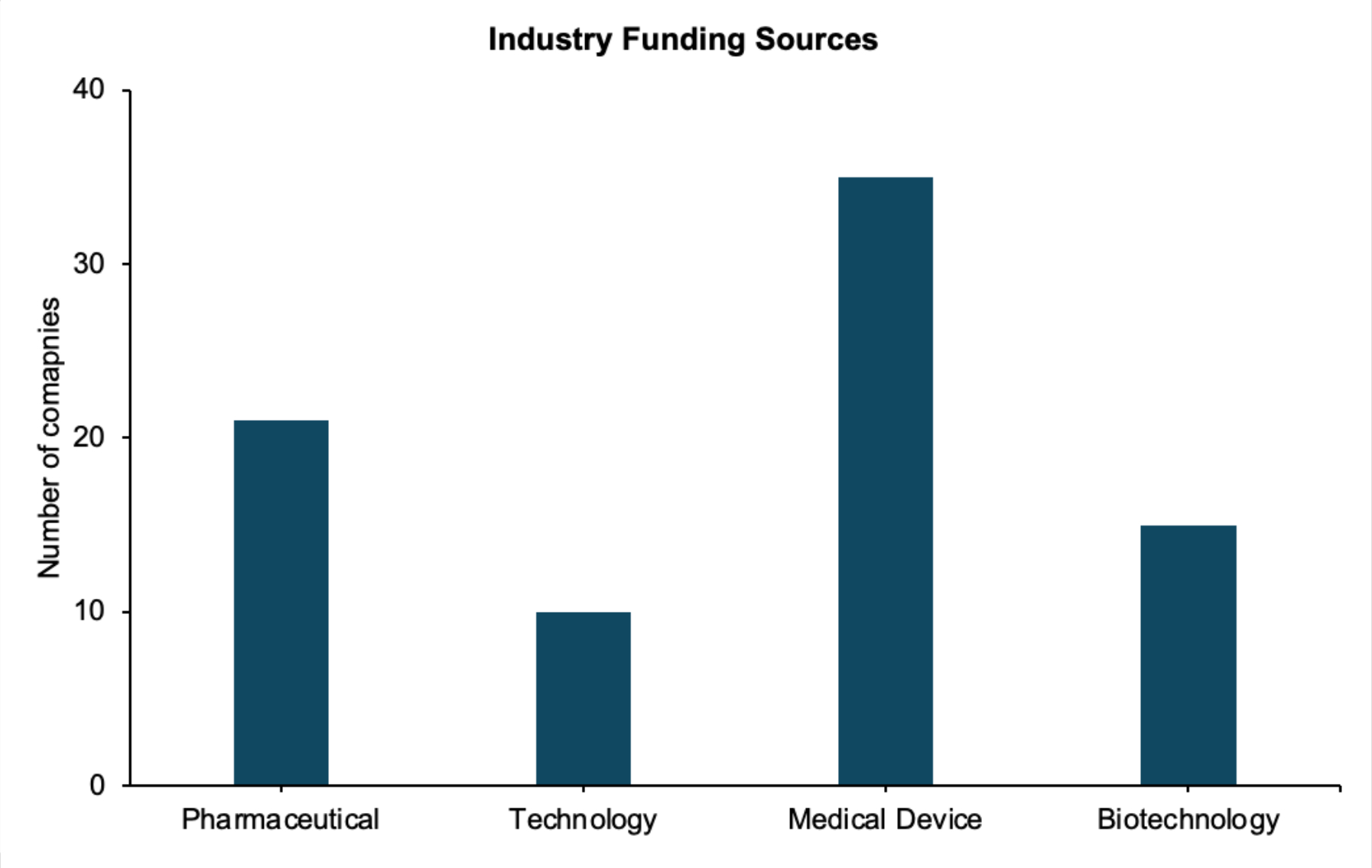

Supplement: Supplementary file 2 [file Image_1.jpeg]

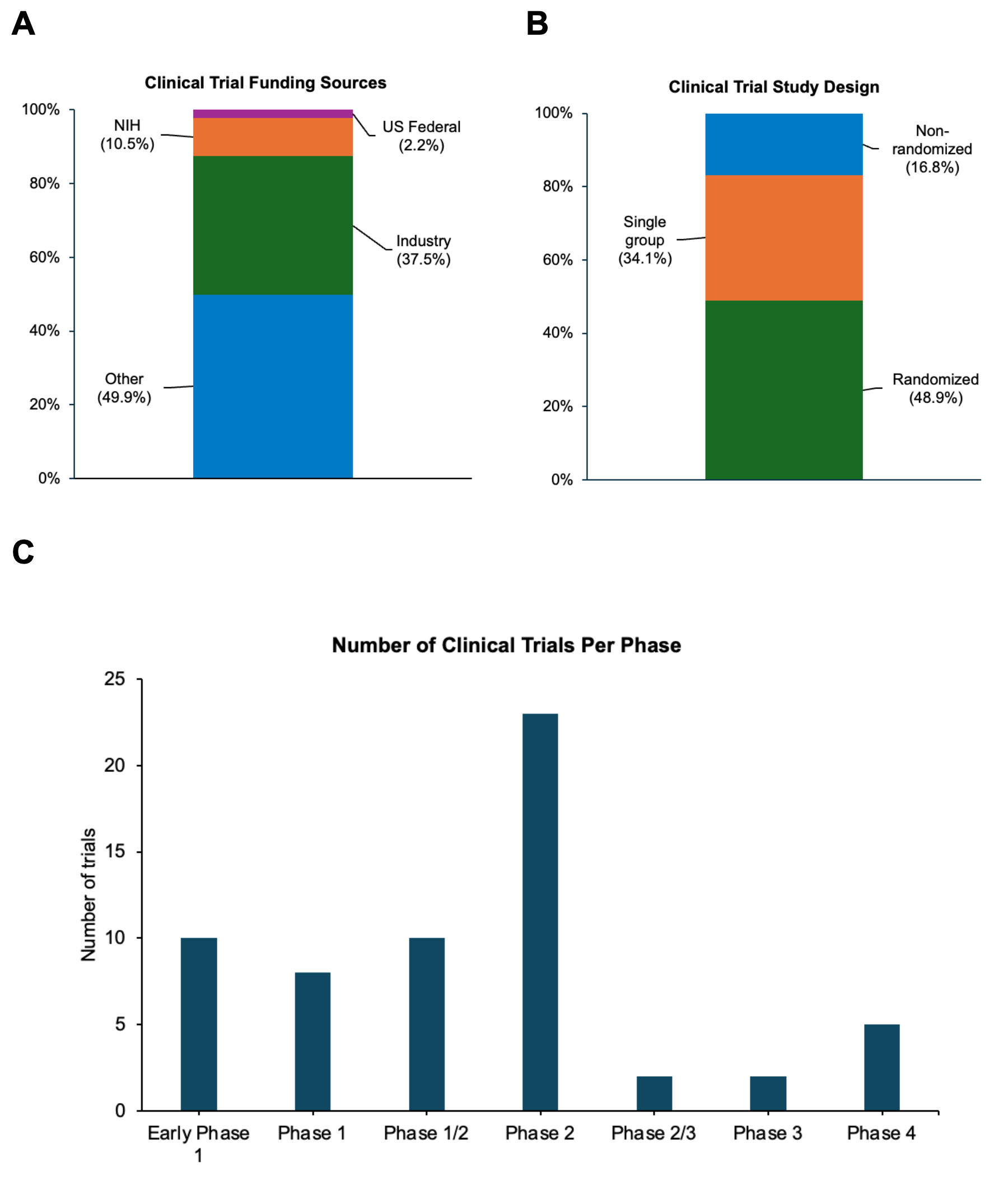

Supplement: Supplementary file 3 [file Image_2.jpeg]

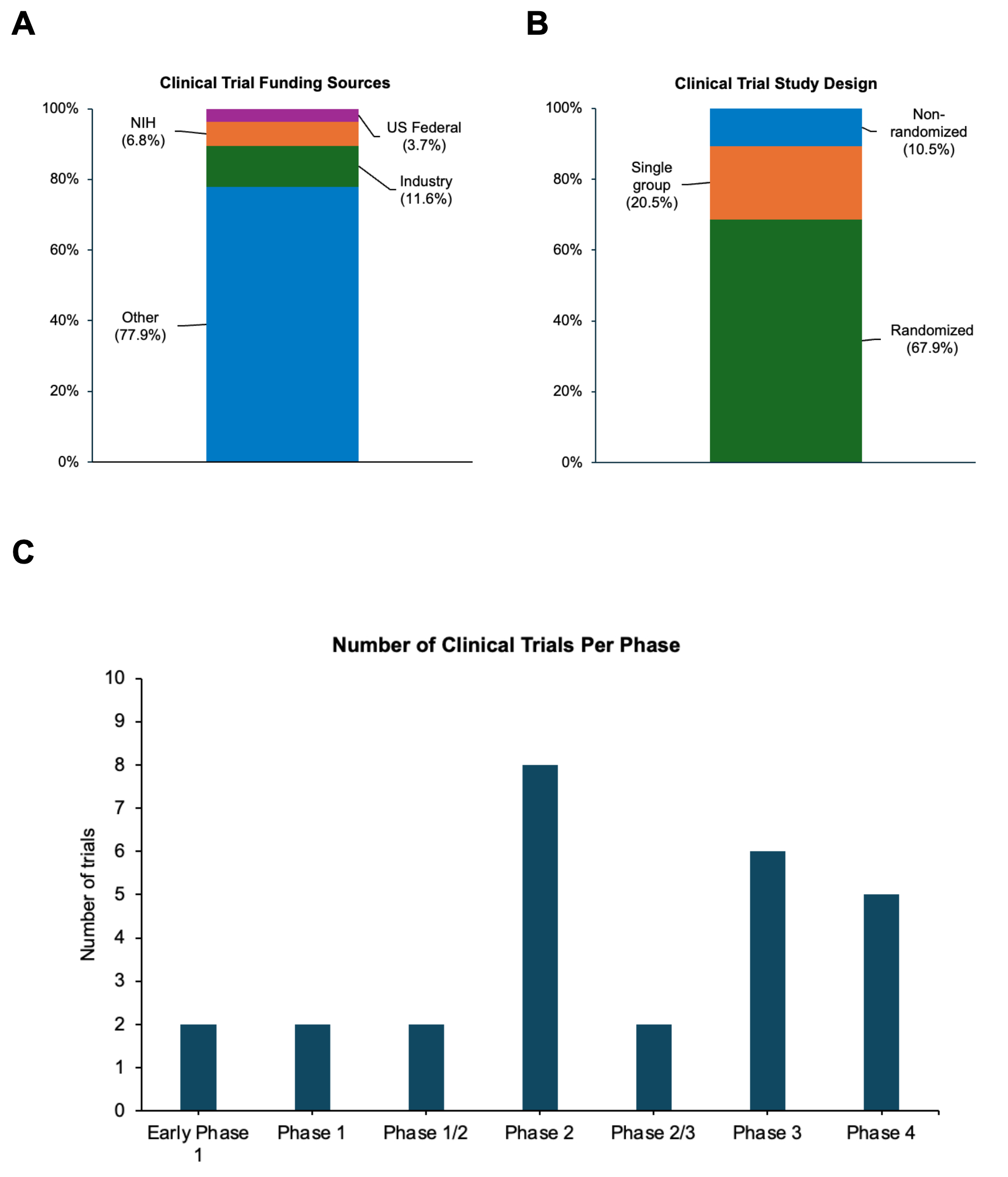

Supplement: Supplementary file 4 [file Image_3.jpeg]

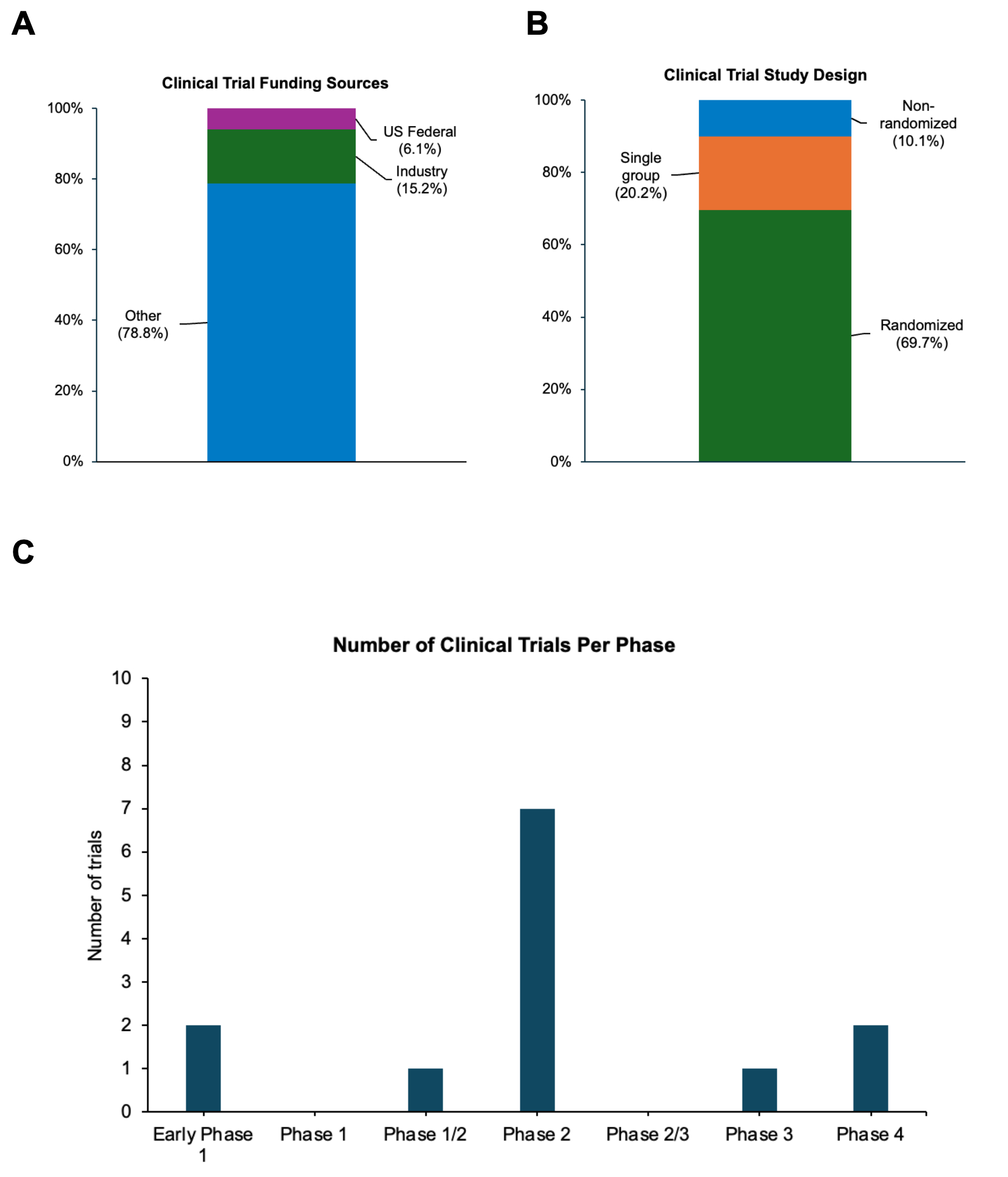

Supplement: Supplementary file 5 [file Image_4.jpeg]

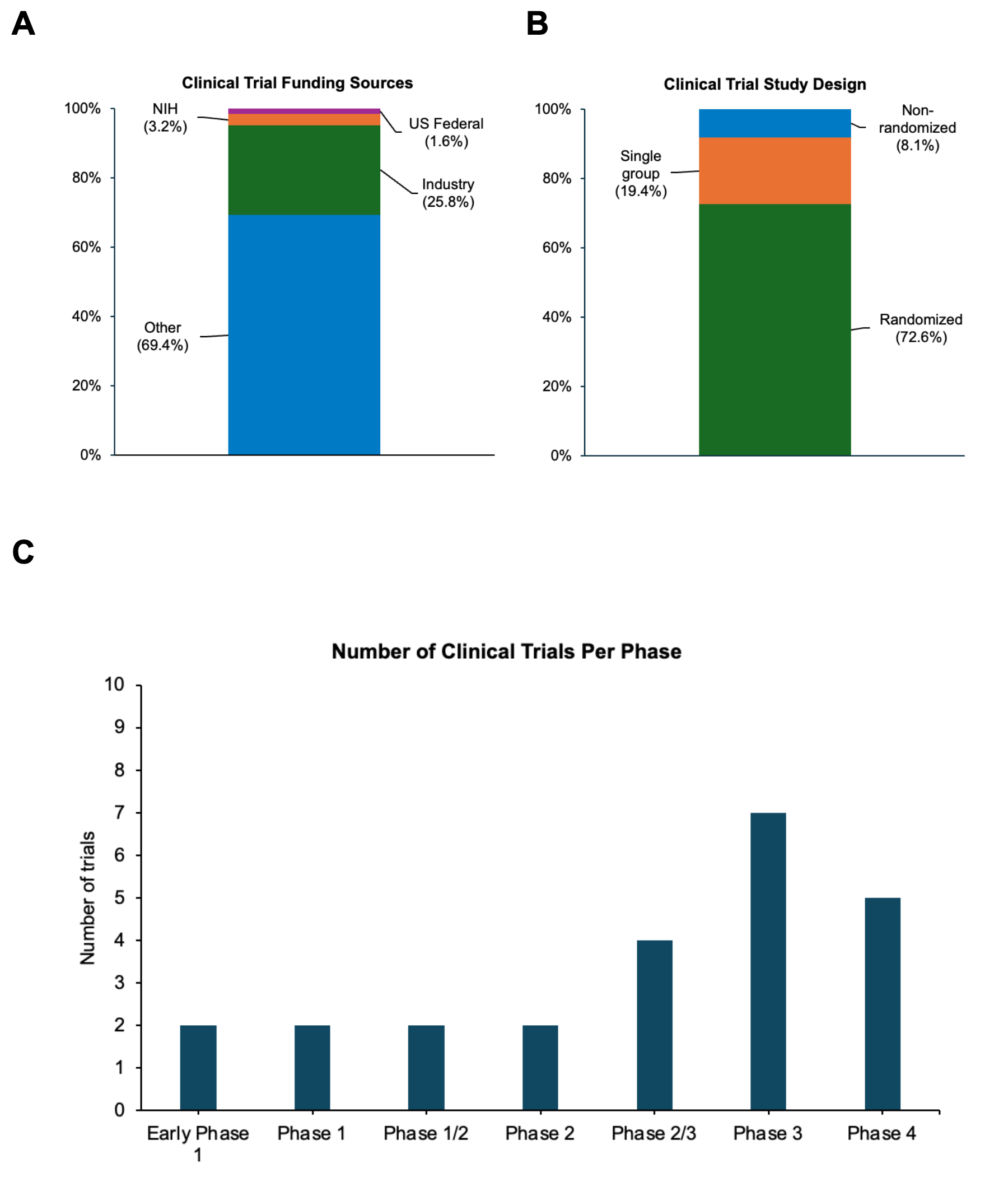

Supplement: Supplementary file 6 [file Image_5.jpeg]

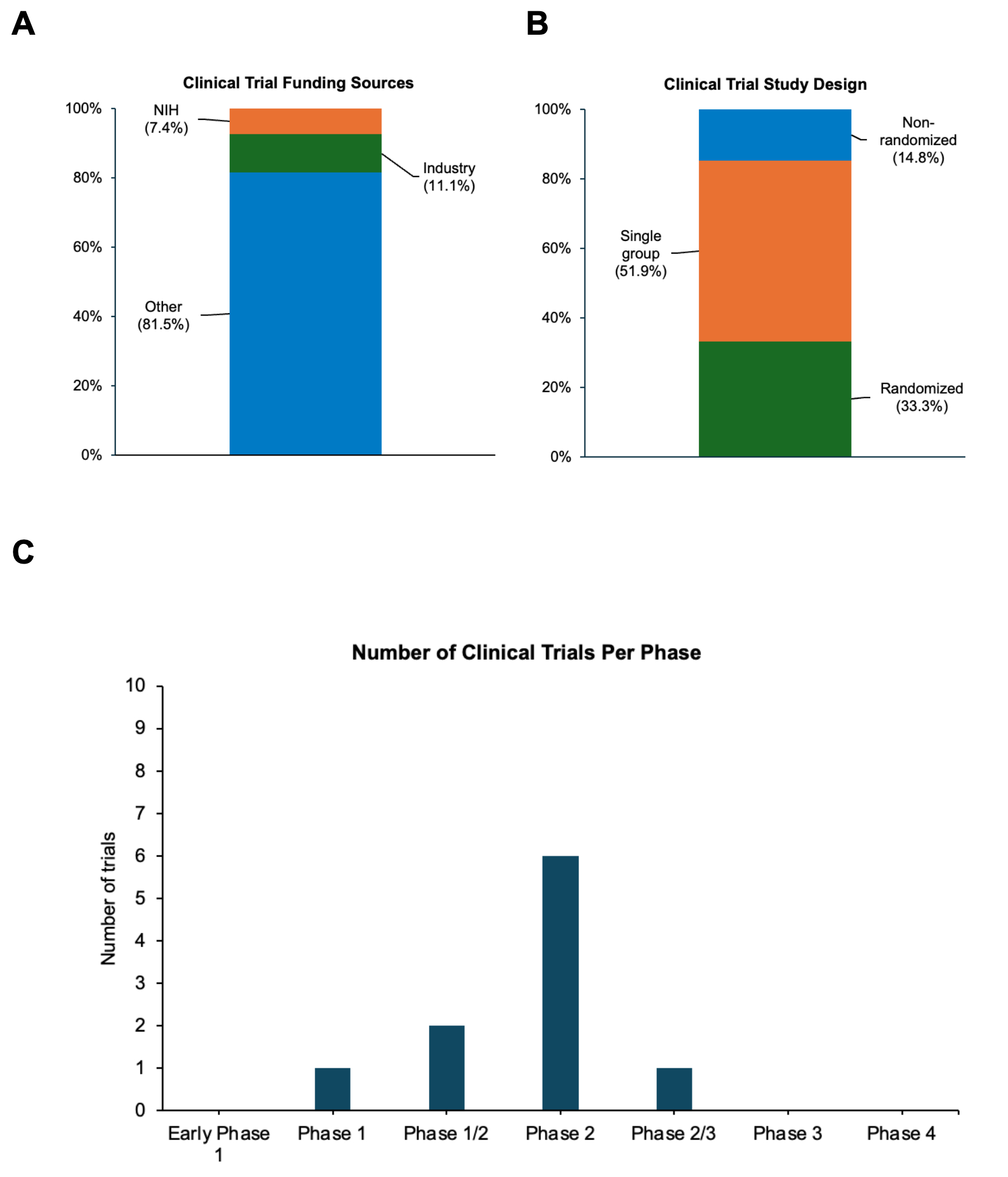

Supplement: Supplementary file 7 [file Image_6.jpeg]

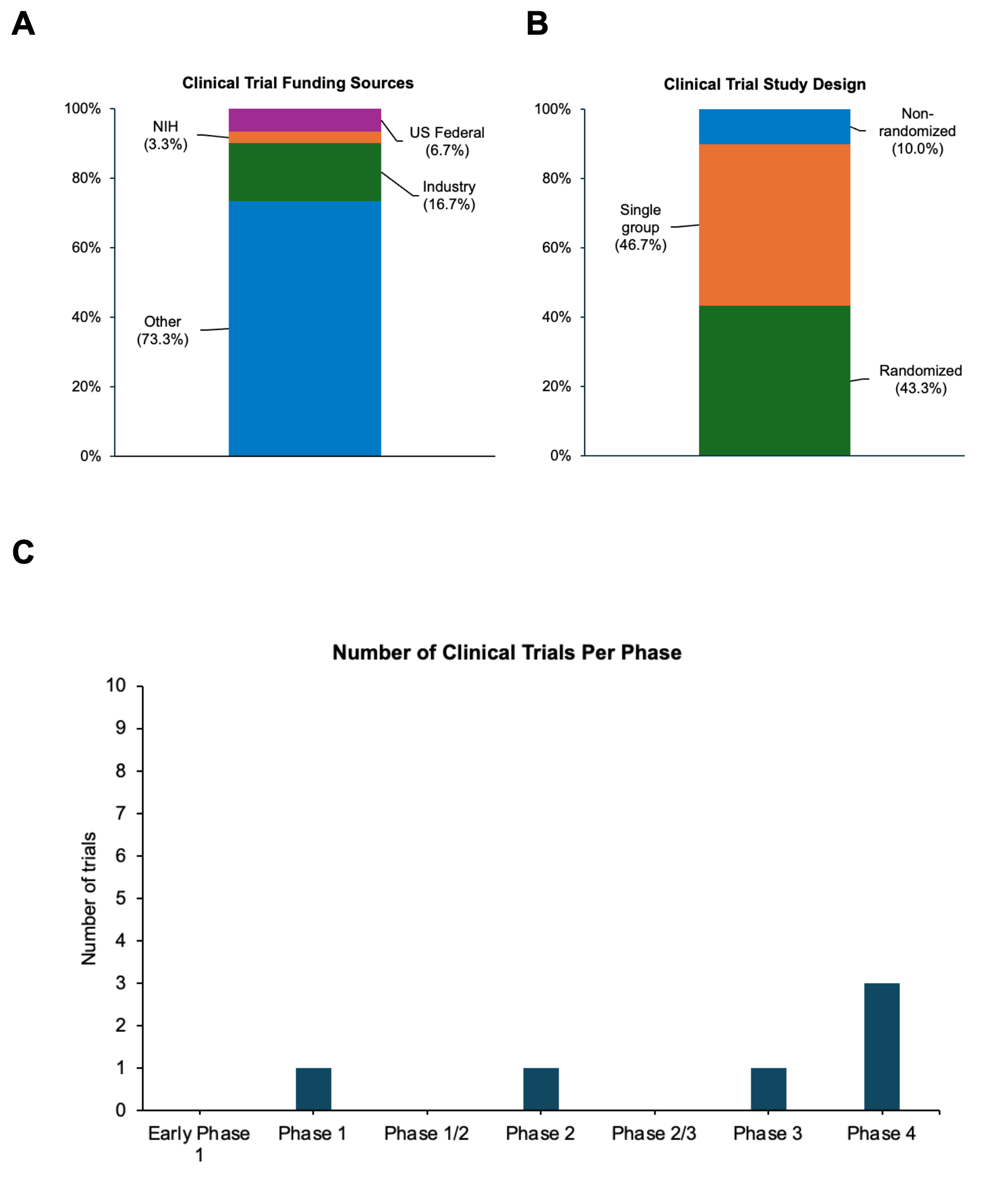

Supplement: Supplementary file 8 [file Image_7.jpeg]
